# RNA-Seq of Gingival Fibroblasts Grown on Collagen Membranes and Hyaluronic Acid

**DOI:** 10.3390/jfb17020057

**Published:** 2026-01-23

**Authors:** Layla Panahipour, Xiaoyu Huang, Reinhard Gruber

**Affiliations:** 1 Department of Oral Biology, University Clinic of Dentistry, Medical University of Vienna, Sensengasse 2a, 1090 Vienna, Austria; 2 Department of Prosthodontics, The Affiliated Stomatological Hospital of Southwest Medical University, Luzhou 646000, China; 3 Department of Periodontology, School of Dental Medicine, University of Bern, 3010 Bern, Switzerland; 4 Austrian Cluster for Tissue Regeneration, 1200 Vienna, Austria

**Keywords:** collagen membrane, hyaluronic acid, gingival fibroblasts, RNA sequencing, biomaterial design, translational research

## Abstract

Purpose: Collagen membranes are widely used biomaterials in periodontal and implant dentistry and can be combined with hyaluronic acid (HA). Although collagen membranes are expected to exhibit bioactive properties and support fibroblast infiltration, their specific impact on fibroblast behavior remains unclear. Methods: To investigate this, human gingival fibroblasts were seeded on collagen matrices—mucoderm^®^, a collagen fleece derived from dermis, and Jason^®^ membrane derived from pericardium—with or without lyophilized HA. Subsequent bulk RNA sequencing was used to assess transcriptional responses. Results: Both mucoderm^®^ and the collagen fleece caused significant transcriptional changes compared with fibroblasts grown on standard tissue culture surfaces and Jason^®^ membrane. These changes included upregulation of *CEMIP*, *STC1*, and *TM4SF1*, and downregulation of *ADM2*, *PSAT1*, and *GPR1*. Notably, the collagen fleece increased expression of extracellular matrix-related genes including *CCN1*, *CCN2*, *COL1A1*, *POSTN*, *SPARC*, *TAGLN*, *FBN2*, *CCDC80*, and *CREB3L1* relative to mucoderm^®^. Additionally, the expression of proteases *MMP3* and *MMP10*, along with detoxification-related genes *MT1E*, *MT2A*, *HMOX1*, and *NQO1*, was relatively decreased. HA coating elevated *IL24* expression in mucoderm^®^, but no similar effect was observed in the collagen fleece. Conclusions: These findings demonstrate that collagen membranes can influence the transcriptome of gingival fibroblasts and suggest that collagen fleece has a stronger effect on extracellular matrix formation than mucoderm^®^. Furthermore, HA coating does not consistently alter fibroblast responses.

## 1. Introduction

Collagen membranes and matrices have become an essential part of clinical practice in dentistry [1], especially for local augmentation in periodontology and implant dentistry [2]. This is based on the well-established technique of guided tissue regeneration, which spatially separates soft tissue from the defect area to create a protected environment for regeneration [3]. Collagen membranes serve as barriers that prevent the rapid growth of soft tissue, particularly fibroblasts. Although initially valued for their cell-occlusive properties and biocompatibility, collagen membranes are now recognized as scaffolds that support osteogenic and immune cell ingrowth [4,5,6,7,8].

Clinically used collagen membranes are mainly derived from porcine tissue [9]. Similar to bone substitutes [10], these membranes undergo processing to remove cellular components and donor tissue impurities [11,12]. Despite this extensive processing, it is essential to preserve the biological and biomechanical properties to prevent immunological reactions while maintaining structural integrity [13]. The collagen-rich extracellular matrix remains structurally intact, potentially retaining part of its original biological activity and cellular components [14,15]. This forms the basis for experimental research aimed at understanding how collagen membranes influence the behavior of immigrating cells.

Histological studies show that collagen membranes act as scaffolds for migrating cells, aiding in new bone formation within their porous structure [6,7]. This property, known as osteoconductivity, describes a material’s ability to promote new bone growth and provoke a cellular response during cell migration. While histological analyses provide valuable insights, they do not offer detailed molecular information, such as transcriptomic changes. The cellular response to collagen membranes can be better understood through transcriptome analysis.

Functional studies have concentrated on how collagen membranes affect precursor cell behavior or specific target gene expression [16,17,18]. RNA sequencing (RNA-seq) now enables comprehensive analysis of cellular responses, capturing the entire transcriptome rather than just individual genes. Recent studies have used RNA-seq to examine responses of various cell types to platelet-rich fibrin [19,20], enamel matrix derivatives [20], and hyaluronic acid (HA) [21]. However, similar analyses of collagen membranes, whether alone or coated with HA, remain lacking, highlighting a notable research gap.

Hyaluronic acid (HA) is a polysaccharide consisting of repeating β (1-4)-glucuronic acid and β (1-3)-N-acetylglucosamine units, which enhances the hygroscopic properties of the extracellular matrix. Cells respond to HA through transmembrane glycoprotein CD44 [22,23], the receptor for hyaluronate-mediated motility [24], and ICAM-1 [25]. Naturally occurring HA is present in synovial fluid [26], skin [27], eyes [28,29], and also in the oral cavity [30], where fibroblasts produce it. Clinically, HA is used for meniscus repair [31,32,33], to treat osteoarthritis of the knee [34] and hip [34], as well as in dentistry [35], including promoting periodontal wound healing, regenerative surgery, peri-implantitis therapy, and alveolar ridge preservation [36].

Combinations of collagen and HA have been explored in various fields, including dentistry. Collagen combined with HA has been proposed as a hemostatic dressing to control bleeding [37,38]. Collagen–HA scaffolds, sometimes with polycaprolactone or growth factors, have been used in vitro [39] or as coatings on titanium discs [40,41]. Collagen membranes with HA delay resorption in diabetic rats [42,43] and enhance bone formation in critical-size defects [44], without disrupting tissue integration or structural degradation [45]. Despite these studies, cellular responses to both collagen membranes and HA coatings are still not fully understood, and this study seeks to clarify them using RNA-seq.

The impact of HA on cellular responses is controversial. In vitro, HA coating can provoke inflammatory responses in oral fibroblasts [46], but RNA-seq shows only moderate effects on cells on tissue culture surfaces [21]. Studies on HA-coated collagen membranes with osteosarcoma cells revealed heterogeneous and weak responses, likely related to focal adhesion and angiogenesis. HA-coated collagen membranes with osteosarcoma cells have shown heterogeneous, weak responses, likely related to focal adhesion and angiogenic signaling [47]. This research motivates further study of commercially available collagen devices containing HA: mucoderm^®^ [48] for soft tissue augmentation, and a collagen fleece^®^ as a hemostatic collagen sponge supporting wound healing, both made from porcine dermis, and Jason^®^ membrane [49] from porcine pericardium for guided bone and tissue regeneration.

The primary goal of this study is to compare the transcriptomic responses of fibroblasts grown on these three collagen devices. The second goal is to clarify how HA influences oral fibroblast responses. This RNA-seq approach offers valuable insights into how fibroblasts respond to the membranes and reveals the effect of HA coating.

## 2. Materials and Methods

### 2.1. Gingival Fibroblasts Grown on Biomaterials

Explant cultures of gingiva from three healthy individuals who provided informed consent were used to isolate gingival fibroblasts. Ethical approval from the Medical University of Vienna’s Ethical Committee (EK #631/2007) is available. These cells were isolated and expanded in Dulbecco’s Modified Eagle Medium (DMEM; Sigma Aldrich, St. Louis, MO, USA) supplemented with 10% fetal calf serum (FCS) and 1% antibiotics (Invitrogen Corporation, Carlsbad, CA, USA). Gingival fibroblasts from three independent donors, all at low passage, were seeded at 30.000 cells/cm^2^ onto mucoderm^®^, collagen fleece (experimental prototype), and Jason^®^ membranes or the same devices added with hyaluronic acid (all botiss biomaterials GmbH, Zossen, Germany). HA coating was performed after chemical processing and prior to the drying process for mucoderm^®^ and Jason^®^ membranes. For the fleece material, HA was added to the chemically processed collagen suspension prior to final freeze-drying. The detailed methodology is confidential. The applied HA exhibits a molecular weight of 1–2 MDa and is from bacterial origin, expressing a low level of bacterial endotoxins. After 24 h of exposure of gingival fibroblasts to the membranes, RNA was isolated and subjected to bulk RNA-seq. All experiments were performed with gingival fibroblasts derived from *n* = 3 independent donors per condition, serving as biological replicates.

### 2.2. Total RNA Isolation, Sequencing, and Data Analysis

Total RNA was isolated from cells grown on the biomaterials, avoiding contamination from cells grown on tissue culture surfaces, using the GeneMATRIX Universal RNA Purification Kit with DNase digestion (EURX, Gdańsk, Poland). Sequencing libraries from total RNA were prepared for the fibroblasts at the Core Facility Genomics, Medical University of Vienna, using the QuantSeq 3′ FWD protocol version 2 with unique dual indices (Lexogen GmbH, Vienna, Austria). Fifteen PCR cycles were used for library preparation, as determined by qPCR following the library prep manual. Libraries were quality-checked on a Bioanalyzer 2100 (Agilent Technologies, Santa Clara, CA, USA) with a high-sensitivity DNA Kit to verify correct insert size and quantified using the Qubit dsDNA HS Assay (Invitrogen, Waltham, MA, USA). Pooled libraries were sequenced on a P2 flow cell on a NextSeq2000 instrument (Illumina, San Diego, CA, USA) in 1x75bp single-end mode. On average, 7 million reads per sample were generated. Reads in FASTQ format were produced using the Illumina bcl2fastq command-line tool (v2.19.1.403) and the Lexogen Idemux tool for optimal demultiplexing of long unique dual indices. Reads were trimmed and filtered with cutadapt version 2.8 to remove polyA tails, discard reads containing N’s, and trim bases with a quality score below 30 from the 3′ ends [50]. After this process, approximately 5 million reads remained. The trimmed FASTQ reads were aligned to the human reference genome GRCh38 with Gencode 29 annotations using STAR aligner [51] version 2.6.1a in 2-pass mode. STAR quantified raw reads per gene. Differential gene expression was analyzed using DESeq2 [52] version 1.22.2. The FASTQ files are available in the GEO repository under the accession number GSE309060.

### 2.3. Principal Component Analysis (PCA), Heat Map, Volcano Plot, and Gene Set Enrichment Analysis

Principal component analysis (PCA) was conducted by R (version 4.5.2), based on normalized expression data to assess sample clustering, donor variability, and overall data quality. The prcomp function was used to project the 21 samples into a two-dimensional space. The first two principal components were captured from the total variance. Samples were plotted using ggplot2(version 4.0.0), with color and shape indicating group and donor origin. Heat map analysis was performed with R (version 4.5.2) from genes which were significantly changed, considering an adjusted *p*-value < 0.05. For volcano plot generation, we used VolcaNoseR, a web-based tool [53]. The up- and down-regulated genes were used for further analysis, with a minimum log2 fold change of 1.5 and a minimum minus log10 significance level of 2.0 (*p* < 0.01) [53]. Protein–protein interaction networks were explored using the STRING database (Search Tool for the Retrieval of Interacting Genes/Proteins, https://string-db.org, access on 13 January 2026). The respective raw data are presented in the Supplement Files.

## 3. Results

### 3.1. Principal Component Analysis and Heat Map of Gene Expression Changes

**Figure 1:**
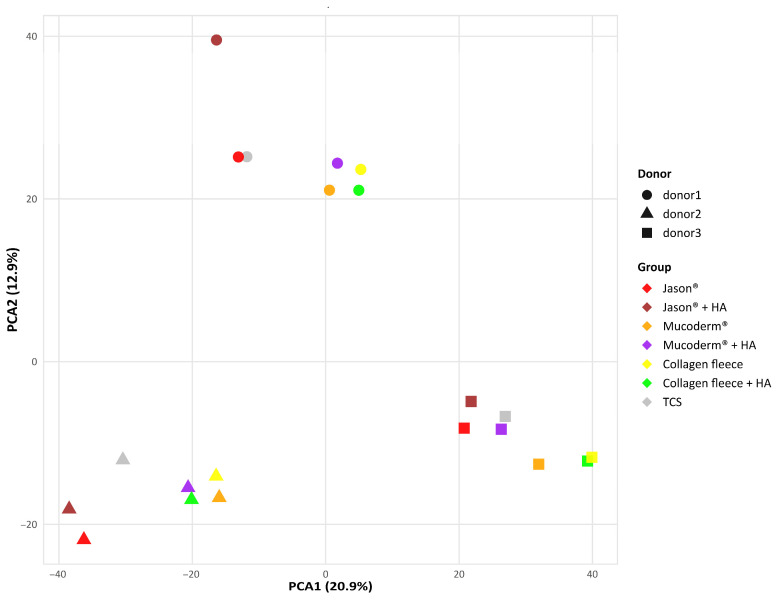
Principal component analysis of differentially expressed genes in gingival fibroblasts grown on collagen membranes. The plot shows the projection of samples onto a two-dimensional space defined by the first and second principal components of the covariance matrix. The input expression levels are normalized log CPM values. PCA highlights donor variation and also the consistent shift in PCA1 and PCA2 caused by Mucoderm^®^ and collagen fleece, regardless of the HA coating.

**Figure 2:**
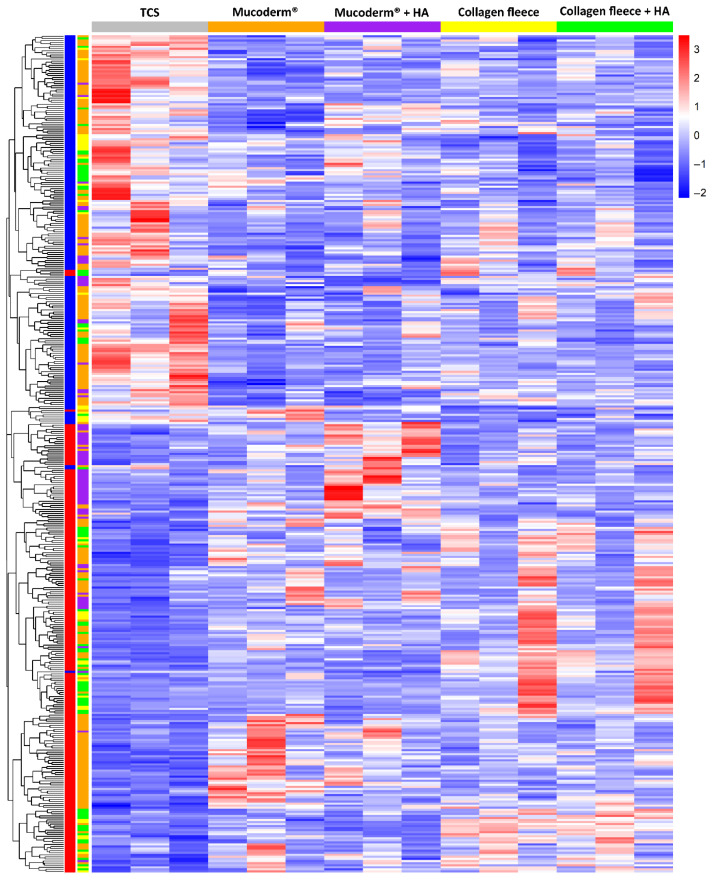
Heat map showing differentially expressed genes in gingival fibroblasts grown on collagen membranes. The dendrogram displays hierarchical clustering of genes based on an adjusted *p* < 0.05, and a log2 fold change of ≥1 or ≤−1. Rows represent genes, while columns represent treatment groups. Red and blue indicate upregulation and downregulation, respectively, relative to untreated cells (tissue culture surface; TCS). The heat map highlights donor variability and a clear differential gene expression pattern when comparing gingival fibroblasts grown on TCS to those grown on Mucoderm^®^ and collagen fleece, with and without HA.

We first performed a PCA to visualize and interpret the relationships between gingival fibroblasts grown on tissue culture surfaces (TCS), mucoderm^®^, collagen fleece, and Jason^®^ membranes, with and without HA (Figure 1). PCA revealed significant variation in the basal transcriptome among the three independent gingival fibroblast preparations. Nevertheless, the response of each fibroblast donor to the different collagen membranes was consistent: mucoderm^®^ caused a clear transcriptomic shift, which was even more pronounced with the collagen fleece, while Jason^®^ membranes elicited little to no change. The effect of HA coating on the PCA was not evident. Consistently, heat map analysis of normalized data showed transcriptomic changes when fibroblasts were grown on mucoderm^®^ and the collagen fleece compared to TCS (Figure 2), but not with Jason^®^ membranes. Additionally, the heat map confirmed that HA coating had no noticeable impact on the transcriptome of gingival fibroblasts. In summary, fibroblasts responded differently to mucoderm^®^ and the collagen fleece, while their expression profiles remained largely similar on TCS and Jason^®^ membranes.

### 3.2. Volcano Plot Comparing TCS with Mucoderm^®^, Collagen Fleece, and Jason^®^ Membrane

**Figure 3:**
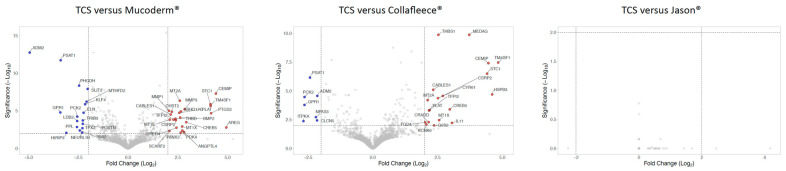
Differentially expressed genes in fibroblasts comparing tissue culture surface with collagen fleece. Volcano plot analysis identified upregulated (red) and downregulated (blue) genes in gingival fibroblasts treated with HA. The annotated dots are data points with the largest (Manhattan) distance from the origin and are above the thresholds indicated by the dashed line. The threshold was set at least a Log2 1.5-fold change and a significance level of *p* = 0.01 (−Log10 of 2). Please note that Jason^®^ membranes behave similarly to tissue culture surfaces (TCS).

Next, we generated volcano plots by applying strict criteria to visualize genes with strong regulation (Table S1). The threshold was set at a log2 1.5-fold change and a significance level of *p* = 0.01 (Figure 3). For mucoderm^®^, 33 genes were upregulated and 27 downregulated compared to fibroblasts grown on TCS. The upregulated genes indicate a reactive stress and remodeling response marked by oxidative defense (*TXNRD1*, *NQO1*, *HSPA1A*, *MT* genes), inflammatory activation (*PTGS2/COX-2*), and ECM remodeling/invasion (*CEMIP*, *THBS1*, *PLAT*, *MMP1*, *MMP3*), along with hypoxia/metabolic adaptation (*STC1*, *ANGPTL4*, *PDK4*, *CYGB*) and repair-associated signaling (*AREG*, *BMP2*, *PTHLH*). Consistently, downregulated genes suggest suppression of an ECM-rich activated mesenchymal program (*COL1A1*, *COL3A1*, *POSTN*, *ELN*, *MXRA5*), accompanied by reduced serine/one-carbon metabolism (*PHGDH*, *PSAT1*, *MTHFD2*, *ALDH1L2*) and decreased stress/proliferation-related signaling (*TRIB3*, *TPX2*).

When fibroblasts were cultured on the collagen fleece, 34 genes were upregulated and 14 downregulated compared to TCS. The upregulated genes indicate a remodeling program characterized by angiogenic signaling (*TM4SF1*, *EDNRB*, *CYR61*), extracellular matrix (*ECM*) remodeling and proteolysis regulation (*CEMIP*, *THBS1*, *TFPI2*, *PLAT*, *NID2*), and a stress/inflammatory response (*SGK1*, *RCAN1*, *NR4A3*, *ARID5A*, *NFIL3*, *IL33*). Consistent with this, there was suppression of an anabolic metabolic program marked by reduced SREBF1-related lipogenesis, decreased serine biosynthesis (*PSAT1*, *PHGDH*), and diminished mitochondrial metabolic flexibility (PCK2). Overall, this pattern suggests a shift from biosynthetically active growth states toward a more metabolically restrained phenotype when cells are grown on the biomaterials rather than on TCS.

A total of 11 genes were commonly upregulated, and 6 genes were downregulated by both mucoderm^®^ and collagen fleece compared to TCS. These include *ABHD17C*, *CABLES1*, *CEMIP*, *CREB5*, *CSRP2*, *EDNRB*, *PLAT*, *STC1*, *TFPI2*, *THBS1*, *TM4SF1*—along with *ADM2*, *EPHB2*, *GPR1*, *PCK2*, *PHGDH*, *PSAT1*, respectively. The most strongly upregulated genes are *CEMIP*, *STC1*, and *TM4SF1*, while *ADM2*, *PSAT1*, and *GPR1* are the most downregulated, potentially serving as marker genes for establishing a bioassay. Conversely, fibroblasts seeded onto Jason^®^ membranes showed gene expression profiles similar to those on TCS (Figure 3). Overall, these findings suggest that mucoderm^®^ and collagen fleece induce transcriptional reprogramming in gingival fibroblasts, whereas Jason^®^ membrane has no significant impact on gene expression compared to TCS.

### 3.3. STRING Analysis Comparing TCS with Mucoderm^®^ and Collagen Fleece

**Figure 4:**
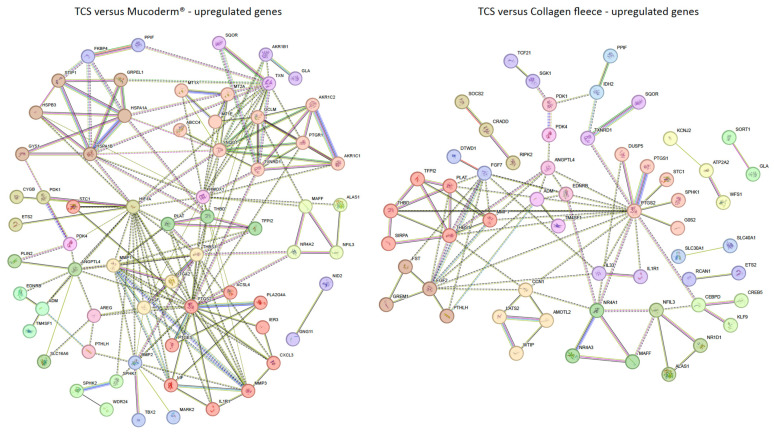
STRING analysis to visualize potential interactions between proteins encoded by up-regulated genes. Data show genes that are upregulated with adjusted *p* < 0.05 and log2 fold change ≥1 or ≤−1 in mucoderm^®^ and collagen fleece compared to a tissue culture surface (TCS). The network was clustered using the Markov Cluster (MCL) algorithm to identify functional modules. Nodes represent proteins, and edges between nodes indicate experimentally confirmed or predicted interactions. The colors of the nodes represent different functional clusters of the proteins.

**Figure 5:**
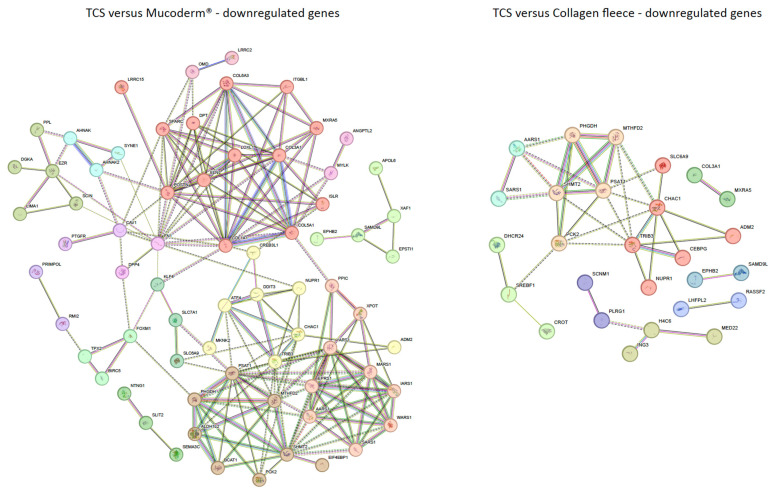
STRING analysis visualizes potential interactions between proteins encoded by down-regulated genes. Data show genes downregulated with adjusted *p* < 0.05, log2 fold change ≥1 or ≤−1 by mucoderm^®^ and collagen fleece compared to a tissue culture surface (TCS). The network was clustered using the Markov Cluster (MCL) method to identify functional modules. Nodes represent proteins, and edges between nodes indicate experimentally confirmed or predicted interactions. The colors of the nodes illustrate different functional clusters of the proteins.

We further conducted a gene ontology (GO) analysis using a less stringent adjusted *p*-value < 0.05 and a log2 fold change threshold of ≥1 or ≤−1 (Table S1). Under these conditions, for mucoderm^®^, 99 genes were upregulated and 114 downregulated compared to a TCS. Figure 4 shows STRING annotation. Mucoderm^®^-induced upregulation was linked to prostaglandin synthesis (red; *PTGS2*, *PTGES*, *PLA2G4A*, *MMP3*, *CXCL3*, *ACSL4*, *LIF*, *IL1R1*, *IER3*, *STC1*), detoxification (salmon; *MT1X*, *MT2A*, *MT1E*, *AKR1C1*, *AKR1C2*, *TXNRD1*, *GCLM*, *NQO1*, *ABCC4*, *PTGR1*), and de novo protein folding (brown; *HSPA1A*, *HSPA1B*, *HSPB3*, *STIP1*, *GYS1*, *GRPEL1*). Conversely, as indicated in Figure 5, downregulated genes were associated with collagen organization (*COL1A1*, *COL3A1*, *COL5A1*, *COL6A3*, *POSTN*, *SPARC*, *ELN*, *ITGBL1*, *DPT*, *ISLR*, *LOXL1*, *LRRC15*, *MXRA5*). 

For the collagen fleece, 98 genes were upregulated and 52 downregulated compared to a TCS. Upregulated genes clustered in pathways linked to negative regulation of fibrinolysis (red; *PLAT*, *TFPI2*, *THBS1*, *MMP1*, *THBD*, *SIRPA*) and prostaglandin synthase activity (salmon; *PTGS2*, *PTGS1*, *STC1*, *SPHK1*, *G0S2*, *DUSP5*). Downregulated genes were enriched in processes related to EIF2AK1 (HRI) response to heme deficiency (red; *TRIB3*, *CHAC1*, *SLC6A9*, *NUPR1*, *ADM2*, *CEBPG*) and L-serine biosynthesis (salmon; *PCK2*, *PHGDH*, *PSAT1*, *SHMT2*, *MTHFD2*). These findings suggest that mucoderm^®^ primarily activates stress-related pathways and extracellular matrix remodeling, whereas the collagen fleece mainly influences fibrinolysis and cellular metabolism compared to a TCS.

### 3.4. Volcano Plot of Gene Expression Changes Caused by HA Coating

**Figure 6:**
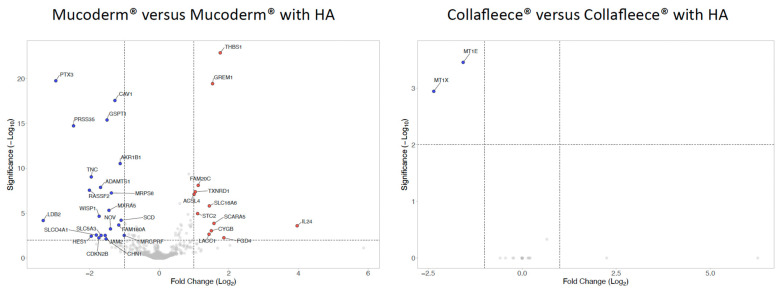
*Differentially expressed genes in fibroblasts caused by HA modification of mucoderm^®^ and collagen fleece*. Volcano plot analysis identified upregulated (red) and downregulated (blue) genes in gingival fibroblasts treated with HA. The annotated dots are data points with the largest (Manhattan) distance from the origin and are above the thresholds indicated by the dashed-line. The threshold was set at a log2 fold change of at least 1.5 and a significance level of *p* = 0.01 (−Log10 of 2). Please note the low impact of HA modification on the gene, which was more pronounced with mucoderm^®^ than with the collagen fleece.

We then evaluated the impact of HA on gene expression, which generally appeared to be minimal (Figure 6, Table S2). In mucoderm^®^, the presence of HA caused the upregulation of 5 genes, including IL24, THBS1, and GREM1, and the downregulation of 13 genes, such as PTX3, PRSS35, and LDB2. In the case of the collagen fleece, HA only reduced the levels of MT1X and MT1E expression. Therefore, when using strict thresholds, HA modification of mucoderm^®^ and collagen fleece had a low and inconsistent effect on the transcriptome of gingival fibroblasts.

### 3.5. Volcano Plot of Gene Expression Changes Comparing Mucoderm^®^ and Collagen Fleece

**Figure 7:**
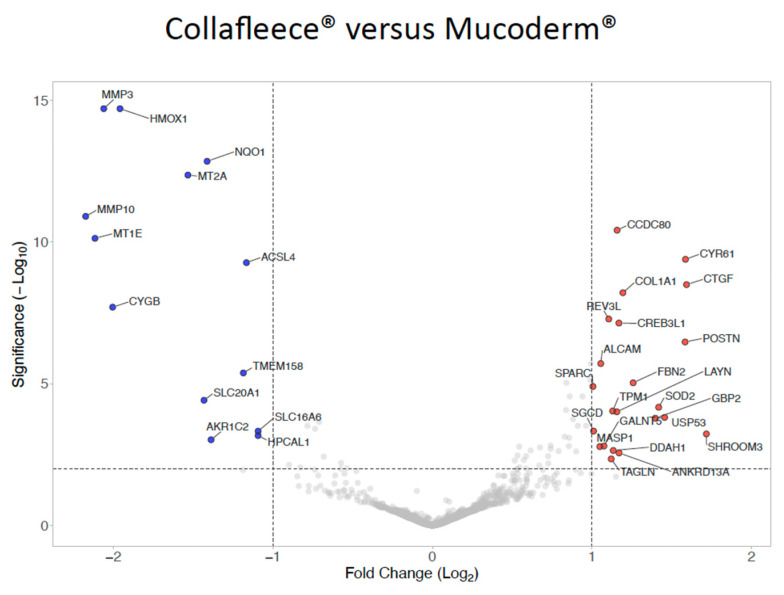
*Differentially expressed genes in fibroblasts grown on collagen fleece versus mucodem^®^*. Volcano plot analysis identified upregulated (red) and downregulated (blue) genes in gingival fibroblasts grown on collagen fleece versus mucoderm^®^. The threshold was set at a log2 fold change of at least 1.5 and a significance level of *p* = 0.01 (−Log10 of 2). Gingival fibroblasts show moderate differential expression changes when grown on the collagen fleece compared to mucoderm^®^.

We finally compared the transcriptomes of gingival fibroblasts grown on collagen fleece versus mucoderm^®^ in the absence of TCS (Figure 7; Table S3). Fibroblasts cultured on collagen fleece had relatively higher transcriptional levels of *POSTN*, *CTGF* (*CCN2*), *CYR61* (*CCN1*), and *SHROOM3*, consistent with enhanced adhesion-dependent mechanotransduction and a wound-healing/ECM-remodeling phenotype. In contrast, mucoderm^®^ showed higher levels of *HMOX1*, metallothioneins (*MT1E*, *MT2A*), *CYGB*, and *MMP3*/*MMP10*, indicating a predominance of oxidative-stress–associated cytoprotective responses coupled to protease-mediated matrix remodeling.

## 4. Discussion

Collagen membranes of xenogeneic origin were initially introduced as biocompatible, degradable barriers that protect soft tissue and maintain the space required for slower bone regeneration, but were not necessarily considered bioactive materials [1,2]. Their main components are fibrillar and non-fibrillar collagens, and their properties can be further improved by adding HA [37,38,39,40,41,42,43,44,45]. Despite extensive processing and multiple decellularization steps, remnants of the original tissue—such as proteins and DNA—may remain within the membrane [14,15]. As a result, collagen membranes not only offer mechanical stability and cell-occlusive functions but also serve as a bioactive matrix that is primarily, though not solely, collagen-based and capable of eliciting cellular responses. These interactions might be especially important during natural healing, when fibroblasts and blood vessels repopulate the scaffold and respond to membrane-intrinsic cues. Therefore, studying how fibroblasts modify their gene expression when attaching to membranes in vitro—and how HA supplementation influences these responses—may provide important mechanistic insights into their potential behavior in vivo.

Comparing fibroblasts grown on a classical TCS with those cultured on three collagen matrices—mucoderm^®^ and a collagen fleece derived from dermis, and the Jason^®^ membrane derived from pericardium—revealed distinct transcriptional signatures. Although TCS is not a clinically relevant control, it provides a highly reproducible standard for in vitro bioassays. After short-term exposure of gingival fibroblasts to collagen membranes or TCS and subsequent RNA sequencing, a panel of genes was consistently up- or downregulated by both mucoderm^®^ and the collagen fleece. These commonly regulated genes are associated with extracellular matrix (ECM) remodeling and angiogenesis (*CEMIP*, *THBS1*, *PLAT*, *TFPI2*), cell cycle regulation and proliferation (*TM4SF1*, *CABLES1*, *CREB5*), signaling and migration (*STC1*, *EDNRB*, *ABHD17C*), and nuclear transport or apoptosis-related processes (*CSEP2*). This conserved transcriptional response indicates fundamental cellular programs triggered when fibroblasts attach to dermis-derived collagenous biomaterials.

Notably, even among dermis-derived biomaterials, fibroblasts exhibited distinct transcriptional profiles, indicating that material processing and ultrastructural properties may be more influential than tissue source in shaping cell phenotype. Under strict filtering conditions, genes that showed increased expression in fibroblasts cultured on the collagen fleece included *POSTN*, *CTGF* (*CCN2*), *CYR61* (*CCN1*), and *SHROOM3*, consistent with enhanced adhesion-dependent mechanotransduction and a wound-healing or ECM-remodeling phenotype. In contrast, fibroblasts grown on mucoderm^®^ displayed higher expression of *HMOX1*, metallothioneins (*MT1E*, *MT2A*), *CYGB*, and *MMP3*/*MMP10*, indicating significant oxidative stress-related cytoprotective responses, combined with protease-driven matrix remodeling. These in vitro findings suggest that the collagen fleece primarily supports ECM synthesis and maintenance, while mucoderm^®^ favors matrix remodeling and degradation.

The underlying reasons for these material-specific transcriptional differences cannot be definitively identified within the scope of this study. Variations in porosity, fibrillar architecture, effective surface area, and manufacturing processes may all influence the observed responses. Notably, fibroblasts cultured on TCS and on the pericardium-derived membrane showed highly similar expression profiles, an unexpected result that calls for further investigation. Overall, a more detailed mechanistic understanding of how collagen-based membranes influence fibroblast gene expression—beyond descriptive transcriptomic changes and including responses such as metallothionein induction—will be necessary and should be linked to clinically relevant functional outcomes in future research.

Metallothioneins (*MT1E*, *MT1X*, *MT2A*) are pleiotropic genes that are highly inducible by heavy metals such as cadmium and zinc [54], for example, in urothelial [55] or endothelial cells [56]. Accordingly, their elevated expression in fibroblasts cultured on mucoderm^®^ may reflect exposure to material-associated stressors, including, but not limited to, trace metals accumulating from animal feed [57] or metals added intentionally to modify membrane properties [58]. Although contamination from animal feed is presumably negligible, collagen membranes have been functionalized with zinc ions, and the presence of residual zinc in the original product cannot be excluded [59]. This possibility is further supported by the broader literature on zinc doping of guided bone regeneration membranes [58]. Notably, HA supplementation reduced *MT1X* and *MT1E* expression, indicating partial dilution or removal of membrane-associated constituents during processing.

Beyond metallothioneins, mucoderm^®^ also induced the aldo–keto reductases AKR1C1 and AKR1C2, key enzymes involved in detoxification of reactive aldehydes and lipid peroxidation products [60]. Their upregulation is commonly linked to oxidative or surface-induced cellular stress rather than to metal exposure per se, suggesting that differences in membrane processing, residual chemicals, or matrix-derived stress signals may contribute to the observed transcriptional signature associated with detoxification.

When considering a less strict threshold for differentially expressed genes, fibroblasts exposed to collagen membranes show transcriptional similarities with cells stimulated by bone allografts [61] and with cells stimulated by lysates from epithelial or carcinoma cell lines [62]. The coordinated upregulation of *AREG*, *CEMIP*, *PLAT*, *MEDAG*, *STC1*, *TM4SF1*, *PTGES*, and *PTGS2* resembles transcriptional programs triggered by damage-associated molecular patterns (DAMPs) released from injured or dying cells. Extracellular histones H2A, H2B, and especially H4 can be present in collagen membranes [14], and may potentially contribute to this conserved stress-response signature [63,64]. Mucoderm^®^ also induced a coordinated stress-adaptation signature characterized by upregulation of *HSPA1A*, *HSPA1B*, *HSPB3*, *STIP1*, *GYS1*, and *GRPEL1*, genes involved in protein folding, mitochondrial quality control, and metabolic adaptation. This transcriptional pattern is typical of early cellular stress responses during sudden environmental changes. Similarly, genes downregulated in fibroblasts grown on mucoderm^®^, including *COL1A1*, *COL3A1*, *COL5A1*, *COL6A3*, *POSTN*, *SPARC*, *ELN*, *ITGBL1*, *DPT*, *ISLR*, *LOXL1*, *LRRC15*, and *MXRA5*, represent a canonical extracellular matrix assembly and stabilization program typical of matrix-producing fibroblasts. Their coordinated suppression suggests a shift from a matrix-synthetic phenotype toward a stress- or remodeling-driven transcriptional state, at least when compared to a TCS.

HA, however, had minimal transcriptional impact. In mucoderm^®^, HA modestly increased *IL24*, *THBS1*, and *GREM1*, while decreasing 12 other genes; whereas in the collagen fleece, HA only reduced *MT1E* and *MT1X*. The latter changes are likely due to dilution or removal of bioactive molecules during coating rather than a direct effect of HA. The upregulation of *IL24* may indicate a context-dependent modulatory response mediated by HA–CD44 signaling [65] rather than a typical inflammatory activation, as no concurrent induction of pro-inflammatory cytokines was observed. Its functional relevance remains uncertain and should be interpreted cautiously. Moreover, this aligns with our previous RNA-seq study showing weak and heterogeneous fibroblast responses to HA alone [21], supporting the growing idea that HA’s clinical effects may be mediated more by biophysical or immunomodulatory mechanisms than by direct fibroblast-driven ECM synthesis. A simple tissue culture substrate—specifically, the culture plate without HA coating—was used as a reference. A collagen membrane-free, HA-only control group was not included in this study. Therefore, the observed transcriptomic changes mainly reflect membrane-driven effects, with HA exerting only minor modulatory influence under the current experimental conditions. Additionally, the lack of strong HA-induced transcriptomic changes does not rule out clinical efficacy but suggests indirect or context-dependent mechanisms.

Several additional limitations should be acknowledged. Principal component analysis and heatmap visualization revealed significant variability between donors in the basal transcriptome of gingival fibroblasts. As a result, donor-specific responses may have been obscured by averaging or, conversely, overly influenced by individual outliers. Future studies should therefore include fibroblasts from a larger and more diverse donor group and use mixed-effects or donor-stratified statistical models to separate baseline donor differences from material-induced effects. Additionally, collecting samples at multiple time points—such as early attachment, matrix deposition, and later remodeling stages—would help determine whether donor-dependent differences persist, diminish, or diverge over time. Ultimately, confirming key transcriptomic signatures at the protein level, as well as in relevant in vivo or ex vivo models, will be crucial to validating the generalizability and translational significance of these findings.

Another limitation is the focus on only the 24 h time point, which mainly captures early cell–material interactions, including attachment-related stress responses and the start of signaling pathways, rather than long-term ECM remodeling or definitive tissue regeneration. However, early fibroblast transcriptional responses have been shown to strongly predict later functional outcomes. Previous in vitro and in vivo studies demonstrate that early induction of ECM-related genes (e.g., *COL1A1*, *CCN1*, *CCN2*, *POSTN*, and *SPARC*), along with regulators of matrix organization and fibrinolysis, is closely linked to subsequent collagen deposition, scaffold integration, and soft-tissue maturation during later stages of wound healing and regeneration. Fibroblasts quickly commit to matrix-producing or matrix-remodeling phenotypes within the first 24–48 h of contact with the biomaterial, and these early transcriptional programs direct downstream protein synthesis, ECM assembly, and tissue organization over days to weeks. Therefore, although the current RNA-seq analysis does not directly evaluate long-term remodeling, it provides mechanistically relevant insights into early molecular signals that lead to and influence later regenerative events.

Concerns may also arise about relying on a single RNA-seq experiment and the absence of experimental validation. RNA sequencing is fundamentally a discovery-driven and hypothesis-generating method. Therefore, this study was intentionally designed as an unbiased transcriptomic screening to characterize early gingival fibroblast responses to clinically used collagen membranes with or without HA. The goal was not to replace functional assays but to create a comprehensive molecular framework to inform future mechanistic and translational research. Although protein-level validation would undoubtedly reinforce the conclusions, the current data should be viewed as early molecular indicators of matrix-modulating behavior rather than definitive functional results.

In summary, human gingival fibroblasts show distinct early transcriptomic responses to different collagen membranes. Mucoderm^®^ and the collagen fleece trigger significant gene expression changes compared to tissue culture surfaces, while the Jason^®^ membrane has minimal impact. The collagen fleece mainly promotes ECM-related gene expression, indicating a matrix-supportive phenotype, whereas mucoderm^®^ primarily activates stress, detoxification, and matrix-remodeling pathways. HA coating has only minor, inconsistent effects, suggesting that fibroblast responses are primarily driven by the collagen membrane rather than HA.

## Data Availability

The original raw sequencing data presented in the study are openly available in the NCBI Gene Expression Omnibus (GEO) repository under accession number GSE309060.

## Supplementary Materials

The following supporting information can be downloaded at: https://www.mdpi.com/article/10.3390/jfb17020057/s1, Table S1 Membranes versus TCS, Table S2 Membranes versus Membranes with HA, Table S3 Collagen fleece versus Mucoderm.
